# CXCL9 Predicts the Risk of Osteoporotic Hip Fracture in a Prospective Cohort of Chinese Men—A Matched Case–Control Study

**DOI:** 10.1002/jbmr.4646

**Published:** 2022-08-17

**Authors:** Quang Tien Phan, Kevin Yiqiang Chua, Aizhen Jin, Christoph Winkler, Woon‐Puay Koh

**Affiliations:** ^1^ Department of Biological Sciences National University of Singapore Singapore Singapore; ^2^ Centre for Bioimaging Sciences National University of Singapore Singapore Singapore; ^3^ Integrative Sciences and Engineering Programme NUS Graduate School, National University of Singapore Singapore Singapore; ^4^ Healthy Longevity Translational Research Programme Yong Loo Lin School of Medicine, National University of Singapore Singapore Singapore; ^5^ Singapore Institute for Clinical Sciences Agency for Science Technology and Research (A*STAR) Singapore Singapore

**Keywords:** BIOCHEMICAL MARKERS OF BONE TURNOVER, MOLECULAR PATHWAYS—REMODELING, CYTOKINES, ENDOCRINE PATHWAYS, OSTEOCLASTS, OSTEOPOROSIS

## Abstract

Recent experimental work has identified CXCL9 as a promoter for the differentiation of osteoclast progenitors into osteoclasts, with resultant bone resorption. However, no human study has validated an association between this chemokine and osteoporosis or fracture risk. We conducted a matched case–control study nested in the prospective, population‐based Singapore Chinese Health Study. Fifty‐five men and 119 women with incident hip fractures, occurring median 6.2 years after blood collection, were matched individually to controls by age at recruitment, sex, and duration of blood storage. Serum chemokines, CXCL9 and CXCL10, were measured using immunoassays. Multivariable conditional logistic regression models that included age at blood collection, body mass index, smoking, and diabetes as covariates were used to estimate odds ratios (OR) and 95% confidence intervals (CI) for association with hip fracture risk. Predictive utility of chemokine for hip fracture risk was examined by comparing area under receiver operating characteristic curves (AUC) between prognostic models with and without the chemokine. Increasing CXCL9 levels were associated with increasing hip fracture risk in men but not in women (*p*_interaction_ = 0.002); comparing extreme quartiles, the OR (95% CI) in the highest quartile was 10.35 (1.90–56.39) in men (*p*_trend_ = 0.002) but 1.46 (0.59–3.60) in women (*p*_trend_ = 0.32). Adding CXCL9 to a prognostic model that already incorporated age and other risk factors improved the AUC (95% CI) from 0.65 (0.55–0.76) to 0.74 (0.65–0.83) for the predictive utility of hip fractures in men but not in women. Conversely, the association between CXCL10 and hip fracture risk was not statistically significant in either sex. © 2022 The Authors. *Journal of Bone and Mineral Research* published by Wiley Periodicals LLC on behalf of American Society for Bone and Mineral Research (ASBMR).

## Introduction

Bone homeostasis requires a balanced activity of various bone cell types, and most importantly, these are the bone‐forming osteoblasts and bone‐resorbing osteoclasts. Osteoporosis occurs when osteoclasts excessively resorb ossified tissues, and this fails to be compensated by adequate bone formation, resulting in frail bones and fractures. In the elderly, hormone reduction during aging causes altered regulation of inflammatory cytokines, which accelerates the bone‐resorbing activities of osteoclasts, leading to higher rates of bone turnover and increased incidence of fractures in older adults.^(^ ^1^, ^2^, ^3^, ^4^ ^)^ Hence, regulatory mechanisms of osteoclast differentiation play an important role in the pathogenesis of osteoporosis.

Chemokines are small signaling cytokines critically involved in many physiological processes, such as host immune responses, wound healing, and bone remodeling.^(^ ^5^, ^6^, ^7^ ^)^ In the context of bone physiology, a subgroup of chemokines that contain a particular arrangement of cysteines in C‐C, C‐X‐C, or CX3C motifs, and their respective receptors, have been shown to regulate bone progenitor cell migration and differentiation by eliciting chemotactic functions.^(^ ^8^, ^9^, ^10^ ^)^ Specifically, two of these chemokines, CXCL9 and CXCL10, have been implicated in different osteoimmuno‐modulating pathways.

First, in vitro studies investigating effects of receptor activator of NF‐κB ligand (RANKL) activation in bone marrow–derived macrophages for osteoclast differentiation, as well as in vivo studies in ovariectomized mouse models for postmenopausal osteoporosis, have shown that CXCL9 and its receptor, CXCR3, are expressed in osteoblasts and osteoclast progenitors, respectively. In these studies, CXCL9, produced by osteoblasts, attracted CXCR3‐positive macrophages and induced osteoclastogenesis. Further, blocking CXCL9‐CXCR3 signaling by ligand antibodies or the CXCR3 antagonist, NBI‐74330, inhibited osteoclast progenitor recruitment and differentiation.^(^ ^11^, ^12^ ^)^ In a medaka fish osteoporosis model suitable for live in vivo imaging, where bone loss was induced by ectopic Rankl expression, we recently elucidated the mechanism behind the critical role of CXCL9 for osteoclast recruitment under experimentally induced osteoporotic conditions.^(^ ^13^, ^14^ ^)^ Another chemokine, CXCL10, binds to the same CXCR3 receptor as CXCL9 and has also been implicated in osteoimmuno‐modulating pathways. For example, in bone metastasis, high CXCL10 serum levels have been shown to promote cancer cell recruitment and osteoclast differentiation, which could in turn lead to osteolytic bone lesions.^(^ ^15^ ^)^

Despite promising experimental data, no human study has validated an association between these chemokines and osteoporosis or fracture risk. Hence, in the present study, we assessed whether levels of CXCL9 and CXCL10 were elevated in human serum samples of older adults who had incident hip fractures in a prospective, population‐based study of older Chinese in Singapore.

## Materials and Methods

### Study population

The current matched case–control study was nested in the Singapore Chinese Health Study, a population‐based prospective cohort designed to investigate the relationship between diet and lifestyle factors and the risk of chronic diseases.^(^ ^16^ ^)^ A total of 63,257 participants (27,959 men and 35,298 women), who were of ages 45 to 74 years between April 1993 and December 1998, were recruited from the two major dialect groups of Chinese adults in Singapore, ie, the Hokkiens, who had originated from Fujian province, and the Cantonese, who had originated from Guangdong province in Southern China. We also restricted our recruitment to residents of public housing flats, where approximately 86% of the population in Singapore resided in during the enrollment period.^(^ ^17^ ^)^ At recruitment, each participant was interviewed in person by interviewers using a structured questionnaire to collect information on demographics, medical history, cigarette smoking, alcohol consumption, physical activity, detailed diet, and other lifestyle factors. This study was approved by the Institutional Review Board at the National University of Singapore, and all enrolled participants gave written informed consent.

During the period from April 1994 to December 1999, a random 3% of the study participants donated blood and single‐void urine specimens for research. Between January 2000 and April 2005, we extended the biospecimen collection to 32,543 participants, which represented a consent rate of about 60% among surviving cohort participants at that time. After collection, the biospecimens were immediately placed on ice during transport to the laboratory and processed within 6 hours for storage in freezers at −80°C. During this period of blood collection, we also updated information on body mass index, smoking, and history of physician‐diagnosed diabetes using structured questionnaires via in‐person or telephone interviews.^(^ ^18^ ^)^

### Cases and controls selection

Hip fracture cases in the Singapore Chinese Health Study cohort were identified via record linkage with a hospital discharge database known as the MediClaim System.^(^ ^19^ ^)^ This database is a nationwide database hosted by the Ministry of Health in Singapore, for the purpose of capturing all patient discharge information submitted by accredited institutions (all the public and private hospitals in the country), regardless of whether there are financial claims or not. Because practically all hip fracture cases will seek medical attention immediately and be hospitalized for clinical care, our case ascertainment through linkage with this comprehensive, nationwide hospital database can be considered complete. To be included in this case–control study, both cases and controls must not have had a hip fracture before giving blood for research. Cases were those with incident hip fracture after blood collection, whereas controls remained free of hip fracture in this study.

Because ours was the first study to examine the association between the serum chemokines and risk of hip fracture, we had first conducted a small pilot study to obtain preliminary risk estimates. At a significance level of 0.05, we estimated that our study had more than 80% power to detect significant risk estimates with a sample size of 50 cases and 50 controls. As such, among the 1630 incident hip fracture cases in the cohort, we randomly chose 55 hip fracture cases in men and 119 cases in women who had given blood for research before their fracture and then found one matching control for each case within the cohort that fulfilled the following matching criteria: age at study recruitment (±3 years), dialect group (Hokkien, Cantonese), date of study recruitment (±2 years), and date of blood collection (±6 months). The selected controls must not have had any fracture at the time of hip fracture for their index cases. Among men, comparing cases in our study (*n* = 55) with the hip fracture patients who were not included in this study (395 men), there were no statistically significant differences in age at fracture, body mass index (BMI), history of ever smoking, and medical history of diabetes. Among the women, compared with the hip fracture patients who were not included in this study (1061 women), the cases in our study (*n* = 119) were younger at fracture (73.3 years versus 74.9 years), more likely to be Cantonese than Hokkien (58.0% versus 45.5%), and less likely to be ever‐smokers (7.6% versus 15.1%), but were otherwise not significantly different in BMI or medical history of diabetes mellitus (Supplemental Table S1).

### Chemokine assays

Enzyme‐linked immunosorbent assay (ELISA) kits were used to determine levels of CXCL9 (Abcam, Cambridge, UK; ab100595) and CXCL10 (Abcam, ab173194) in serum samples, following the manufacturer's instructions. Briefly, serum samples were diluted four times with diluent provided in the kit (for CXCL9) or used undiluted (for CXCL10). Serum samples and serial dilutions of human recombinant proteins were incubated in 96‐well plates precoated with antibodies detecting CXCL9 and CXCL10, respectively. Plates were washed and incubated with tetramethylbenzidine (TMB) solution provided by the kit for 30 minutes in the dark and with gentle shaking. STOP solution containing 0.15 M sulfuric acid provided by the kit was added to terminate the color development, and optical densities (ODs) were read at 450 nm. Standard curves were generated using OD values from serial dilutions of human recombinant proteins. Serum concentrations were obtained by plotting OD values of serum samples against the standard curves. All measurements were done in duplicates. The intra‐assay coefficients of variation (CVs) for the OD readouts from the ELISA assays were 7.63% for CXCL9 and 4.16% for CXCL10, both within the recommended range of less than 10%.

### Statistical analysis

All the participants included in our study had complete data. Student's *t* tests (for continuous variables) and chi‐square tests (for categorical variables) were used to compare baseline characteristics between the hip fracture cases and controls. The distributions of serum chemokines were markedly skewed with long right tails, and they were corrected, to a large extent, by transforming the original values to their natural logarithms (Supplemental Fig. S1). To account for our matched study design, fixed‐effects linear models, adjusted for age at blood draw (years, continuous), BMI (kg/m^2^, continuous), history of smoking (never, ever), and history of diabetes mellitus (yes, no) were used to compare the mean chemokine levels between cases and controls in the whole cohort and then in men and women, separately.

We applied conditional logistic regression, which accounted for our matched study design, to estimate the odds of hip fracture associated with the second, third, and fourth quartiles of each serum chemokine, using the first quartile as reference, first in the whole cohort, and then in separate analyses for men and women. These sex‐specific quartiles were included as indicator/dummy variables in our models, and their cut‐off values were based on the distribution of the serum chemokine among controls within each sex. When assessed as continuous variables, the risk estimates quoted were based on every 10% increment in serum chemokine levels (pg/mL). The age‐adjusted model included age at blood draw (years, continuous), whereas the overall adjusted model additionally included BMI (kg/m^2^, continuous), history of smoking (never, ever), and history of diabetes mellitus (yes, no); the last factor has been shown to significantly increase risk of hip fracture in our study population.^(^ ^20^ ^)^ The magnitude of the associations was assessed by odds ratios (OR) and their corresponding 95% confidence intervals (CI). We tested for linear trends by entering the median value of each quartile as a continuous variable into our regression models, and we assessed the statistical significance of any interaction between sex and CXCL9 levels by including the product term between these two factors of interest in our fully adjusted models.

We used receiver operating characteristic (ROC) analysis to evaluate the incremental value of CXCL9 for the prediction of osteoporotic hip fracture in men and women separately. Three prognostic models, each composed of an (unconditional) logistic regression model, were fitted to distinguish between cases and controls. Model 1 accounted for our matched study design by incorporating the risk factors that were used to match cases to controls (age at blood draw and dialect group). Model 2 included the variables in Model 1, plus other risk factors for hip fracture, ie, BMI (kg/m^2^, continuous), history of smoking (never, ever), and history of diabetes mellitus (yes, no). Lastly, in addition to all the factors in Model 2, Model 3 included log‐transformed CXCL9 levels. The area under the ROC curve (AUC) for each of these models was then calculated to provide an assessment of the predictive performance of each model, and the resultant values were compared against each other using an algorithm described by DeLong and colleagues.^(^ ^21^ ^)^

All statistical analysis was conducted using Stata/SE 14.2 (StataCorp LLC, College Station, TX, USA) and SAS Version 9.2 (SAS Institute, Inc., Cary, NC, USA). All reported *p* values were two‐sided, and *p* < 0.05 was considered statistically significant.

## Results

In our study, the mean time interval from blood draw to the occurrence of hip fractures was 6.3 (standard deviation [SD] 2.9) years among the 55 cases in men and 5.8 (SD 2.9) years among the 119 cases in women. Median time from blood draw to hip fracture was 6.2 years for all the cases combined. The mean age of cases at the occurrence of hip fracture was 75.4 (SD 7.8) years in men and 73.3 (SD 6.9) years in women. Compared with their respective controls who remained free of hip fractures, the men who developed hip fractures had a lower mean BMI and were more likely to be smokers, whereas the women who developed hip fractures were more likely to have a history of diabetes mellitus (Table 1).

**Table 1:** Baseline Characteristics of Individuals Who Developed Hip Fractures (Cases) and Those Who Remained Free of Hip Fractures (Controls), the Singapore Chinese Health Study

Characteristics	Cases	Controls	*p* Value^a^
**Men**			
No. of subjects	55	55	
Age (years) at blood draw, mean (SD)	69.1 (7.3)	68.8 (7.0)	0.83
Dialect group			1.00
Hokkien, *n* (%)	25 (45.5%)	25 (45.5%)	
Cantonese, *n* (%)	30 (54.5%)	30 (54.5%)	
Body mass index (kg/m^2^), mean (SD)	21.5 (3.7)	22.9 (2.7)	0.032
History of smoking			0.083
Never smokers, *n* (%)	19 (34.5%)	28 (50.9%)	
Ever smokers, *n* (%)	36 (65.5%)	27 (49.1%)	
History of diabetes mellitus, *n* (%)	8 (14.6%)	8 (14.6%)	1.00
**Women**			
No. of subjects	119	119	
Age (years) at blood draw, mean (SD)	67.5 (6.6)	67.2 (6.7)	0.69
Dialect group			1.00
Hokkien, *n* (%)	69 (58.0%)	69 (58.0%)	
Cantonese, *n* (%)	50 (42.0%)	50 (42.0%)	
Body mass index (kg/m^2^), mean (SD)	23.1 (3.9)	23.6 (3.9)	0.29
History of smoking			0.37
Never smokers, *n* (%)	106 (89.1%)	110 (92.4%)	
Ever smokers, *n* (%)	13 (10.9%)	9 (7.6%)	
History of diabetes mellitus, *n* (%)	35 (29.4%)	10 (8.4%)	<0.001

^a^ Two‐sided *p* value was derived from *t* test for continuous variables and from chi‐square test for categorical variables.

Table 2 shows the adjusted geometric means of the chemokines in serum samples collected from participants who subsequently developed hip fractures (cases) and those who remained free of hip fractures (controls). When analyzed as a cohort, cases had significantly higher serum levels of CXCL9 compared with controls (*p* = 0.009). However, in sex‐specific analysis, it was found that only male cases had significantly higher levels of CXCL9 when compared with their control counterparts (*p* < 0.001); among women, there was no statistically significant difference in serum CXCL9 levels between cases and controls (*p* = 0.47). Unlike our findings for CXCL9, there was no significant difference in serum levels of CXCL10 between cases and controls in either combined or sex‐stratified analysis (*p* > 0.18).

**Table 2:** Adjusted Geometric Means (95% Confidence Intervals) of Serum Chemokines in Individuals Who Developed Hip Fractures (Cases) and Those Who Remained Free of Hip Fractures (Controls), the Singapore Chinese Health Study

Serum chemokines	Geometric mean (95% CI)^a^		
	Cases	Controls	*p* Value
**All subjects**	174	174	
CXCL9 (pg/mL)	2591.1 (2343.9, 2864.3)	2131.3 (1928.0, 2356.0)	0.009
CXCL10 (pg/mL)	73.2 (68.5, 78.3)	71.8 (67.2, 76.8)	0.70
**Men**	55	55	
CXCL9 (pg/mL)	2905.2 (2546.0, 3315.1)	2000.0 (1752.7, 2282.2)	<0.001
CXCL10 (pg/mL)	68.1 (62.0, 74.7)	61.9 (56.4, 68.0)	0.18
**Women**	119	119	
CXCL9 (pg/mL)	2407.7 (2109.9, 2747.5)	2240.2 (1963.2, 2556.4)	0.47
CXCL10 (pg/mL)	74.8 (68.5, 81.7)	77.9 (71.3, 85.1)	0.55

^a^ Adjusted for age at blood draw (years, continuous), body mass index (kg/m^2^, continuous), history of smoking (never, ever), and history of diabetes mellitus (yes, no).

The associations between increasing quartile levels of serum chemokines and the risk of developing a hip fracture are shown in Table 3. In fully adjusted models, participants with higher quartiles of CXCL9 levels showed a positive association with an increased risk of developing hip fractures only among men (*p*_trend_ = 0.002) but not among women (*p*_trend_ = 0.32). Compared with men with the lowest quartile of CXCL9 levels, men with the highest quartile of CXCL9 levels had an OR (95% CI) of 10.35 (1.90, 56.39) for the risk of hip fracture; in contrast, the OR (95% CI) comparing extreme quartiles in women was 1.46 (0.59, 3.60). In a fully adjusted model, every 10% increment in serum CXCL9 levels was associated with a significant OR (95% CI) of 1.17 (1.06, 1.29) for the risk of hip fracture in men. In comparison, we found no significant associations between increased CXCL9 levels and risk of hip fracture in women (OR [95% CI] of 1.02 [0.98, 1.06]; *p* for interaction between sex and CXCL9 = 0.002). Finally, in both men and women, we did not find any statistically significant associations between CXCL10 and the risk of hip fracture.

**Table 3:** Odds Ratios (OR) and 95% Confidence Intervals (CI) for the Associations Between Serum Chemokines and the Risk of Developing Hip Fracture, the Singapore Chinese Health Study

	Quartile 1	Quartile 2	Quartile 3	Quartile 4	*p*_trend_	Per 10% increment^b^
***Men***						
**CXCL9 (pg/mL), median (IQR)**	1170.0 (851.3, 1404.7)	1747.0 (1590.7, 1855.3)	2378.7 (2226.7, 2592.7)	3652.0 (3094.0, 4422.0)		2385.0 (1656.0, 3415.3)
Cases/controls	5/14	8/14	13/14	29/13		55/55
Age‐adjusted OR (95% CI)	Ref.	2.25 (0.31, 16.54)	5.90 (1.00, 34.99)	11.18 (2.03, 61.74)	0.002	1.18 (1.07, 1.31)
Overall adjusted OR (95% CI)^a^	Ref.	1.81 (0.21, 15.76)	6.12 (0.98, 38.24)	10.35 (1.90, 56.39)	0.002	1.17 (1.06, 1.29)
**CXCL10 (pg/mL), median (IQR)**	46.0 (42.2, 49.7)	56.1 (54.2, 57.3)	65.5 (61.2, 69.4)	90.7 (80.7, 109.1)		61.3 (53.7, 74.6)
Cases/controls	11/14	6/14	23/14	15/13		55/55
Age‐adjusted OR (95% CI)	Ref.	0.52 (0.14, 1.97)	3.28 (0.98, 10.99)	2.40 (0.68, 8.42)	0.15	1.08 (0.96, 1.22)
Overall adjusted OR (95% CI)^a^	Ref.	0.43 (0.09, 2.11)	3.59 (0.98, 13.14)	2.71 (0.70, 10.55)	0.18	1.10 (0.97, 1.26)
***Women***						
**CXCL9 (pg/mL), median (IQR)**	1101.3 (811.3, 1267.3)	1688.7 (1559.3, 1890.0)	2538.7 (2266.7, 2896.0)	5329.7 (4052.3, 9008.0)		2231.0 (1492.0, 3660.0)
Cases/controls	24/30	21/30	39/30	35/29		119/119
Age‐adjusted OR (95% CI)	Ref.	0.92 (0.40, 2.14)	1.66 (0.76, 3.61)	1.67 (0.74, 3.75)	0.15	1.02 (0.98, 1.05)
Overall adjusted OR (95% CI)^a^	Ref.	0.83 (0.33, 2.10)	1.79 (0.78, 4.14)	1.46 (0.59, 3.60)	0.32	1.02 (0.98, 1.06)
**CXCL10 (pg/mL), median (IQR)**	48.1 (41.8, 54.7)	65.5 (62.7, 70.7)	83.6 (79.0, 89.3)	119.2 (109.3, 174.6)		71.7 (58.0, 97.8)
Cases/controls	37/30	35/30	16/30	31/29		119/119
Age‐adjusted OR (95% CI)	Ref.	0.90 (0.44, 1.84)	0.42 (0.19, 0.97)	0.80 (0.39, 1.64)	0.54	0.99 (0.94, 1.04)
Overall adjusted OR (95% CI)^a^	Ref.	0.77 (0.35, 1.67)	0.45 (0.18, 1.11)	0.71 (0.32, 1.58)	0.48	0.98 (0.93, 1.04)

^a^ Adjusted for age at blood draw (years, continuous), body mass index (kg/m^2^, continuous), history of smoking (never, ever), and history of diabetes mellitus (yes, no).

^b^ For every 10% increment in serum chemokine levels (pg/mL, continuous).

**Fig. 1:**
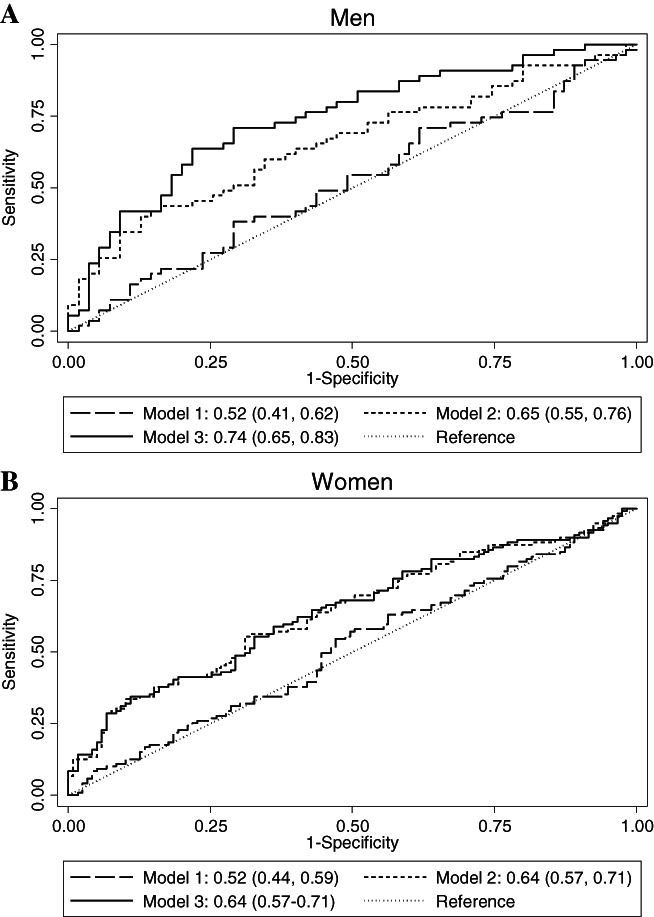
Receiver operating characteristic (ROC) curves demonstrating the incremental utility of incorporating CXCL9 into prognostic models for the prediction of osteoporotic hip fracture in men (*A*) and women (*B*). Model 1: Logistic regression model incorporating age at blood draw (years, continuous), dialect group (Hokkien, Cantonese). Model 2: All factors in model 1 + BMI (kg/m^2^, continuous), history of smoking (never, ever), history of diabetes mellitus (yes, no). Model 3: All factors in model 2 + CXCL9 [log(pg/mL), continuous].

ROC curves illustrating the ability of various prognostic models to predict osteoporotic hip fracture were plotted in men (Fig. 1*A*) and women (Fig. 1*B*) separately. In men, adding CXCL9 as a continuous variable to a model, which already contained age at blood draw, dialect group, BMI, smoking, and history of diabetes, further increased the AUC (95% CI) from 0.65 (0.55, 0.76) in Model 2 to 0.74 (0.65, 0.83) in Model 3 for the prediction of hip fracture. In contrast, among women, CXCL9 had negligible incremental predictive value when added to Model 2.

## Discussion

To the best of our knowledge, this is the first longitudinal study to prospectively investigate the association between serum chemokines, CXCL9 and CXCL10, and the risk of osteoporotic hip fracture in humans. Our findings revealed higher serum levels of CXCL9 in the pre‐fracture blood samples of men with subsequent hip fractures compared with their non‐fracture controls. In contrast, there was no such difference in CXCL9 serum levels between cases and controls in women. Correspondingly, we also showed that CXCL9 had a positive association with the risk of hip fracture only in men. Furthermore, our results demonstrated that serum CXCL9 improved the prediction of osteoporotic hip fracture in men.

Although we did not measure bone mineral density (BMD) in this study, the latter is used to diagnose osteoporosis in clinical practice. Interestingly, however, according to a World Health Organization report, the majority of osteoporotic fracture patients have had BMDs above the diagnostic criteria for osteoporosis (ie, a *T*‐score > −2.5).^(^ ^22^ ^)^ This indicates limitations of using BMD alone to identify those at risk of fractures. Thus, there is intense interest to find biomarkers that can serve as independent diagnostic and prognostic indices or be used as a complementary indicator to BMD for osteoporotic fractures.^(^ ^23^ ^)^ Naturally, bone biomarkers produced from the bone remodeling process, including bone formation biomarkers, bone resorption biomarkers, and regulators of bone turnover, are likely candidates as predictors of osteoporotic fracture risk.^(^ ^24^ ^)^ Future work needs to determine whether CXCL9 predicts fracture independently from BMD and can thus be a possible risk marker in addition to BMD.

Our previous work in a medaka fish osteoporosis model has shown that CXCL9‐CXCR3 signaling is required and sufficient for osteoclast progenitor recruitment to osteoporotic lesion sites.^(^ ^14^ ^)^ Using live in vivo imaging, we demonstrated that osteoblast‐derived Cxcl9l, the CXCL9 ortholog in fish, functioned to attract Cxcr3.2‐positive macrophages to bone matrix. After reaching the matrix, these cells dynamically interacted with bone‐lining osteoblasts and started to differentiate into functional osteoclasts, which resorbed bone matrix. Excessive bone resorption and osteoporotic lesions in this animal model were prevented by treatment with the CXCR3 antagonists AMG 487 and NBI‐74330.^(^ ^14^ ^)^ This confirmed a critical role for the Cxcl9l‐Cxcr3.2 axis for osteoclast recruitment under experimentally induced osteoporotic conditions. In the present study, our novel data showed that CXCL9 could be used as a predictor of hip fracture risk in older men. Hence, our results open the possibility that early interventions targeting CXCL9 or CXCL9‐CXCR3 signaling could be beneficial in preventing hip fractures in older men.

Although our finding in men is supported by our previous experimental work, the null finding in women is unexpected. Postmenopausal women have a higher prevalence of osteoporosis and greater incidence of fracture than older men. The apparent sex discrepancy in fracture risk has been attributed to sex differences in bone geometry, the dominant sex hormones, and the pattern of sex hormone changes during aging.^(^ ^25^ ^)^ Compared with men, women have a lower peak bone mass during adulthood, after which women undergo a rapid decline of estrogen during menopause that leads to accelerated loss of primarily trabecular bone, followed by a relatively slow phase of cortical bone loss.^(^ ^26^, ^27^ ^)^ Interestingly, studies in ovariectomized (OVX) mice showed that estrogen deficiency upregulated the levels of CXCL9 in bone marrow and that CXCL9 neutralization in vivo alleviated bone loss after ovariectomy.^(^ ^12^ ^)^ Although women may have a higher lifelong exposure to estrogen than men, it is known that older men could have higher levels of circulating estradiol than postmenopausal women, predominantly owing to increased aromatase activity in adipose tissues, with subsequent conversion of testosterone to estradiol in men.^(^ ^28^ ^)^ Hence, because estrogen negatively regulates CXCL9 expression and secretion in osteoblasts, this could explain why the blood levels of CXCL9 were lower in older men (mean of 2000 pg/mL) than in older postmenopausal women (mean of 2240 pg/mL) without hip fracture in the present study. Furthermore, because all women in the present study were at an advanced stage of menopause during blood collection (mean age of 67 years), it is plausible that the lack of a significant difference in CXCL9 levels could be explained by similarly low levels of estrogen between cases and controls in postmenopausal women. Further studies are needed to understand how CXCL9 levels may be regulated differentially by sex hormones in men and women.

Although CXCL9 and CXCL10 share a common receptor, CXCR3, in triggering cell migration, our null finding in CXCL10 for both men and women suggests that these two chemokines may have different effects on bone physiology. Interestingly, although CXCL10 was shown to facilitate trafficking of CXCR3‐expressing cancer cells to the bone, which then augmented its own production and promoted osteoclastic differentiation, this was in the context of osteolytic bone metastasis.^(^ ^15^ ^)^ Finally, when comparing extreme quartiles of CXCL10 in men, although the odds ratio of 2.71 for the risk of hip fracture did not meet the threshold for statistical significance in our study (Table 3), this risk estimate could possibly reach statistical significance in future studies with larger sample sizes.

The strength of the present study is the collection of exposure data and blood specimens before hip fracture occurrence in a prospective cohort, thus overcoming temporal bias and recall bias in typical retrospective case–control studies. One limitation of the study is that the results were based on a single time‐point collection of blood samples. However, since the serum samples were collected before fractures, such misclassifications are generally nondifferential in nature and may result in an underestimation of the true effect size of the chemokine on hip fracture risk. Another limitation is the lack of measurements on BMD; hence we were unable to examine whether the chemokines were independent of BMD on risk of hip fracture.

In conclusion, the present study demonstrated, for the first time, that CXCL9 could have incremental predictive value for the risk of osteoporotic hip fracture in older men but not in women. While our epidemiologic findings are supported by experimental data that have provided the mechanistic pathway for the role of CXCL9 in regulating osteoclast recruitment, further studies are needed to confirm the validity of our findings and determine their generalizability to other study populations. Furthermore, the underlying biological mechanisms that limit our findings to men but not women require further investigation.

## Disclosures

All authors state that they have no conflicts of interest.

## Author Contributions

**Quang Tien Phan:** Data curation; investigation; methodology. **Kevin Yiqiang Chua:** Data curation; formal analysis; writing – review and editing. **Aizhen Jin:** Data curation; formal analysis; writing – review and editing. **Christoph Winkler:** Conceptualization; funding acquisition; project administration; supervision; writing – original draft; writing – review and editing. **Woon‐Puay Koh:** conceptualization; funding acquisition; project administration; supervision; writing – original draft; writing – review editing.

### Peer Review

The peer review history for this article is available at https://publons.com/publon/10.1002/jbmr.4646.

## Supporting information


**Appendix S1.** Supporting information

## Data Availability

Data are available on reasonable request due to privacy/ethical restrictions.
