# Supplementary material for: CXCL9 Predicts the Risk of Osteoporotic Hip Fracture in a Prospective Cohort of Chinese Men—A Matched Case–Control Study
Source: J Bone Miner Res. 2022 Aug 17;37(10):1843–9. doi: 10.1002/jbmr.4646 (PMC9804917; doi:10.1002/jbmr.4646)
Supplement: Supplementary file 1 — Appendix S1. Supporting information [file JBMR-37-1843-s001.pdf]

Supplementary Figure 1: Histograms of CXCL9 and CXCL10 serum levels.

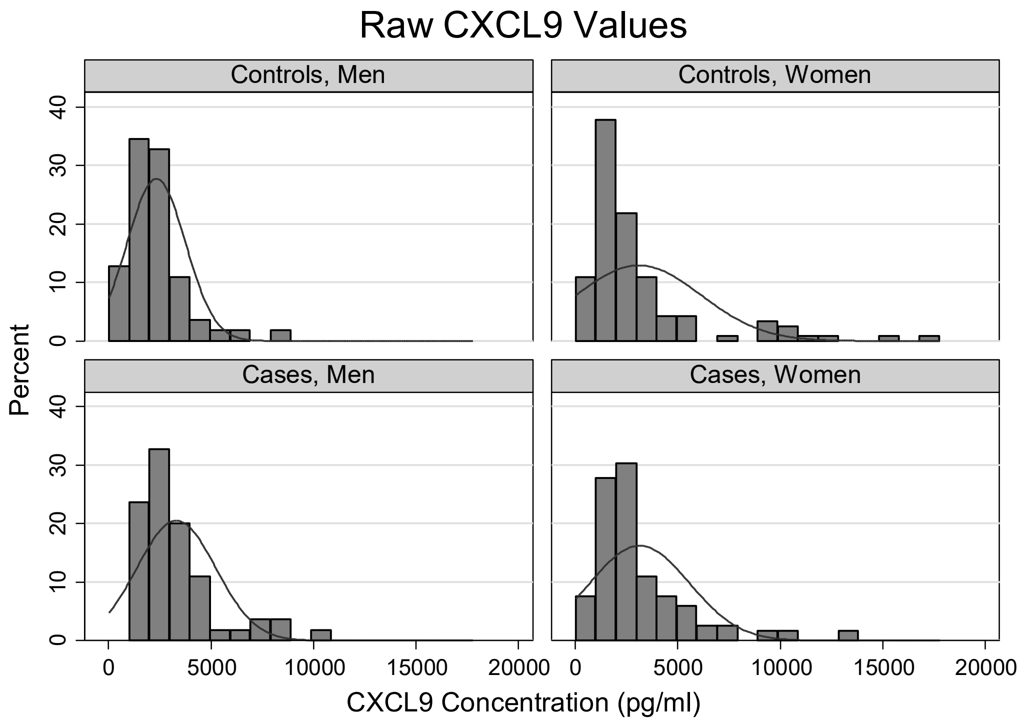

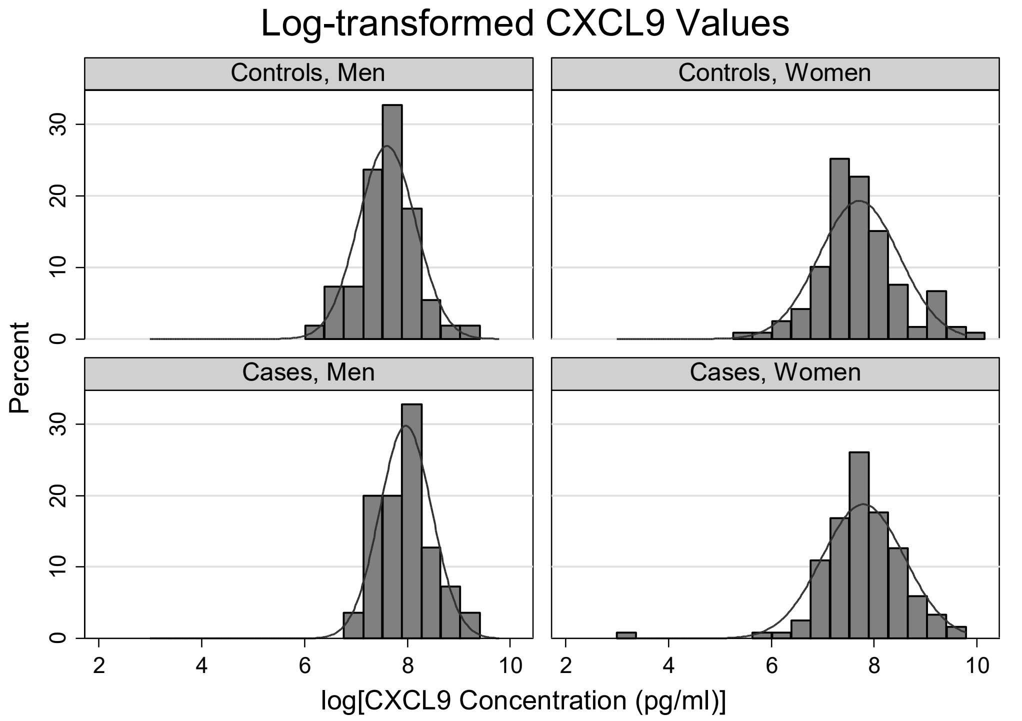

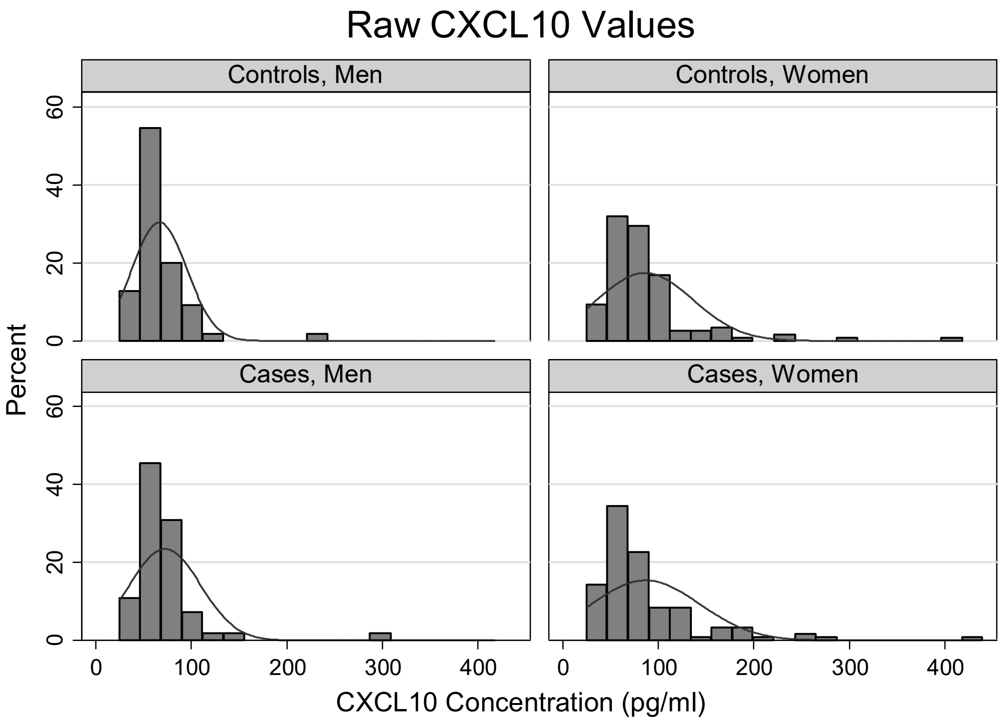
Raw CXCL10 Values
Controls, Men
Controls, Women
Cases, Men
Cases, Women
Percent
0
20
40
60
0
100
200
300
400
CXCL10 Concentration (pg/ml)

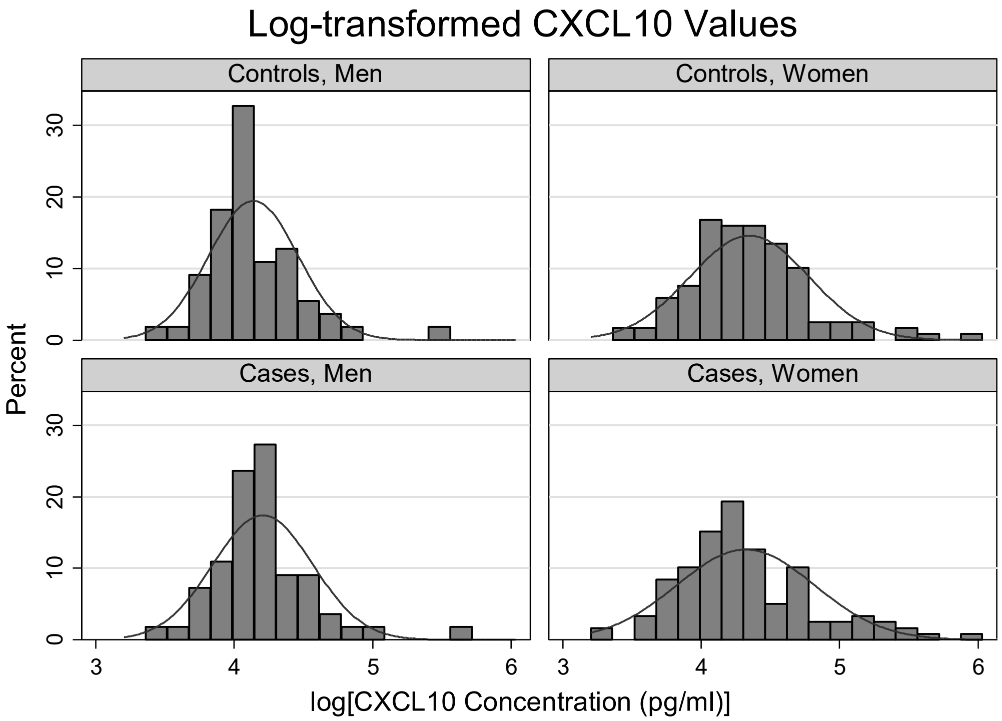
Log-transformed CXCL10 Values
Controls, Men
Controls, Women
Cases, Men
Cases, Women
Percent
0
10
20
30
3
4
5
6
log[CXCL10 Concentration (pg/ml)]

Supplementary Table 1. Baseline characteristics [n (%) or mean (SD)] of hip fracture cases in the study and those not in the study.

**Characteristics**	**Cases in study**	**Cases not in study**	***P*-value ***
**Men**			
Number of subjects	55	395	
Age at fracture, mean (SD)	75.4 (7.8)	73.6 (8.1)	0.116
Dialect group			
Hokkien, n (%)	30 (54.5)	249 (63.0)	0.224
Cantonese, n (%)	25 (45.5)	146 (37.0)	
Body mass index (kg/m^2), mean (SD)	22.8 (3.4)	22.3 (2.8)	0.363
History of smoking			
Never smokers, n (%)	25 (45.5)	129 (32.7)	0.061
Ever smokers, n (%)	30 (54.5)	266 (67.3)	
History of diabetes mellitus, n (%)	5 (9.1)	53 (13.4)	0.370
**Women**			
Number of subjects	119	1061	
Age at fracture, mean (SD)	73.3 (6.9)	74.9 (7.3)	0.024
Dialect group			
Hokkien, n (%)	50 (42.0)	578 (54.5)	0.010
Cantonese, n (%)	69 (58.0)	483 (45.5)	
Body mass index (kg/m^2), mean (SD)	23.5 (3.3)	23.2 (3.2)	0.285
History of smoking			
Never smokers, n (%)	110 (92.4)	901 (84.9)	0.026
Ever smokers, n (%)	9 (7.6)	160 (15.1)	
History of diabetes mellitus, n (%)	17 (14.3)	229 (21.6)	0.063

* 2-sided *P*-value was derived from *t* test for continuous variables and from chi-squared test for categorical variables
